# COVID-19: A call for awareness or mandatory vaccination even in pandemics?

**DOI:** 10.7189/jogh.11.03005

**Published:** 2021-01-30

**Authors:** Yen Jun Wong, Shaun Wen Huey Lee

**Affiliations:** 1 School of Pharmacy, Monash University Malaysia, Bandar Sunway, Selangor. Malaysia; 2 Asian Centre for Evidence Synthesis in Population, Implementation and Clinical Outcomes (PICO), Health and Well-being Cluster, Global Asia in the 21st Century (GA21) Platform, Monash University Malaysia, Selangor, Malaysia; 3 Gerontechnology Laboratory, Global Asia in the 21st Century (GA21) Platform, Monash University Malaysia, Selangor, Malaysia; 4 School of Pharmacy, Taylor’s University Lakeside Campus, Jalan Taylors, Selangor, Malaysia

The World Health Organization (WHO) declared COVID-19 as pandemic on 11th March 2020 [1]. To date, more than 42 million people have tested positive for COVID-19, and over one million lives have been sacrificed to the disease worldwide [2]. As part of an effort to break the chain of the severe acute respiratory syndrome coronavirus 2 (SARS-CoV-2) transmission, mandatory lockdown and travel restrictions including physical confinement were implemented globally. This unprecedented pandemic has directly and indirectly affected the health and well-being of society catastrophically. On top of the physical debilitation suffered by patients, the deterioration of mental health has pervaded all levels of communities.

Childhood immunization plays an important role in preventing life-threatening communicable diseases such as polio, diphtheria, pertussis, measles, and tuberculosis [3]. Every country has a similar but distinctive childhood immunization program to ensure the protection of children’s health against preventable diseases following vaccine utilization. However, the enforcement on movement restriction coupled with the sense of fear and anxiety of contracting the highly infectious SARS-CoV-2 virus might pose alarming challenges to routine childhood immunization.

Amidst COVID-19 pandemic and the global lockdown, WHO, United Nations Children's Fund (UNICEF), and Centers for Disease Control and Prevention (CDC) had reported potential disruption to the childhood immunization program worldwide [4,5], especially in low and middle-income countries (LMICs), due to (1) supply chain issues with transport limitations, (2) restriction of travel to follow the vaccination schedule for schools and parents, (3) health care centres prioritizing COVID-19 response, and (4) parents who refuse to present their children at health clinics for vaccination as they are anxious about the risk of COVID-19 infection to their children [4,6].

In Malaysia, the National Immunization Program was first introduced in the 1950s, aiming to tackle preventable communicable diseases in children [3]. While Malaysia has improving vaccination coverage over the years with an estimated childhood vaccination coverage of more than 90% in 2019 [7], a drop of vaccination uptake in measles, mumps and rubella (MMR) vaccination was reported by about 60% to 70% since the introduction of lockdown in March this year. The decline was similarly seen in varicella vaccination by a range of 41% to 83% from March to May [8]. While the reasons for such drop are unclear, these could be due to the service disruptions, economic hardships or even people reluctance to leave home to visit health facilities. Indeed, the message that public has been urged to stay at home provides little assurance that public health facilities remain open for routine care.

**Figure:**
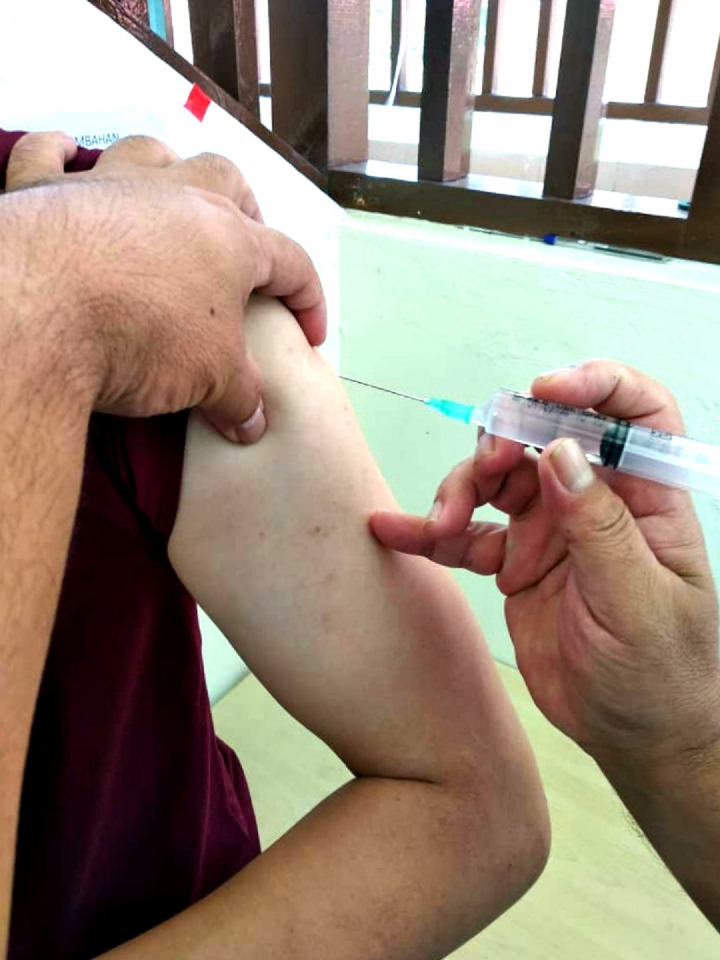
Photo: From the author’s own collection (used with permission).

While parents may have hesitancy in adhering to their children’s immunization schedule in fear of exposing their children to SARS-CoV-2 virus, the effort in sustaining good childhood vaccination coverage must not slack. Unfortunately, instead of strengthening legislation and enforcement to ensure that the people adhere to their children’s immunization schedule, the Malaysian Government recently announced that the country will not mandate childhood vaccination by law enforcement [9]. This is deeply concerning since childhood immunization is important as a cost-effective approach to protect the community from vaccine preventable diseases that could lead to disability and fatality.

As such, it is essential that regular and strict surveillance on childhood vaccination especially the new-borns by health care workers is implemented during this pandemic. Education is important to instil the correct knowledge about vaccination among parents. The conscientious initiative in promoting childhood vaccination can be seen from the efforts of the Ministry of Health, the Malaysian Paediatric Association (Mpaeds), and Malaysian Public Health Physicians' Association (PPPKAM). During this COVID-19 pandemic, the Ministry of Health ensures that precautionary measures are strictly adhered to while attending parents and children who are present at the health clinics for child vaccination, to dampen the anxiety and paranoia from parents [10]. A community education initiative by Immunise4life, which provides childhood vaccination information on the internet portal, has been actively interacting with communities and attentively responding to queries about vaccination from the public.

More community and online-based interactive educational interventions can be planned and conducted, to overcome the misconception about childhood immunization, while improving the health literacy of society towards vaccination. In primary care settings, community pharmacists could play an important role in advocating childhood vaccination uptake through education to enhance public awareness towards the significance of routine childhood vaccination.

Despite high coverage of childhood vaccination, in 2019, Malaysia reported a measles outbreak as well as the resurfacing of polio since 1992. Taking this as an important lesson, the re-emergence of vaccine preventable diseases should not be a repeated catastrophe to our health system, where resources could be much utilized to tackle potential evolving infectious diseases. All authorities from the Government and Private sectors should work hand-in-hand to encourage routine childhood immunization, in order to protect the health and well-being of our children, and to be freed from vaccine preventable diseases.
